# Proprioceptive Disturbance in Chronic Neck Pain: Discriminate Validity and Reliability of Performance of the Clinical Cervical Movement Sense Test

**DOI:** 10.3389/fpain.2022.908414

**Published:** 2022-07-06

**Authors:** Ulrik Röijezon, Gwendolen Jull, Christian Blandford, Anna Daniels, Peter Michaelson, Petros Karvelis, Julia Treleaven

**Affiliations:** ^1^Division of Health, Medicine and Rehabilitation, Department of Health, Education and Technology, Luleå University of Technology, Luleå, Sweden; ^2^School of Health and Rehabilitation Sciences, University of Queensland, Brisbane, QLD, Australia; ^3^Department of Informatics and Telecommunications, University of Ioannina, Arta, Greece

**Keywords:** cervical spine, image analysis, laser pointer, neck pain, proprioception, sensorimotor, tracking task, video recording

## Abstract

Chronic neck pain is associated with sensorimotor dysfunctions, which may develop symptoms, affect daily activities, and prevent recovery. Feasible, reliable, and valid objective methods for the assessment of sensorimotor functions are important to identify movement impairments and guide interventions. The aim of this study was to investigate the discriminative validity of a clinical cervical movement sense test, using a laser pointer and an automatic video-based scoring system. Individuals with chronic neck pain of idiopathic onset (INP), traumatic onset (TNP), and healthy controls (CON) were tested. Associations between movement sense and neck disability were examined and the repeatability of the test was investigated. A total of 106 participants (26 INP, 28 TNP, and 52 CON) were included in a cross-sectional study. *Acuity, Speed, Time*, and *NormAcuity* (i.e., normalized acuity by dividing acuity with movement time) were used as outcome measures. ANOVAs were used for group comparisons and Pearson correlations for associations between movement sense variables and neck disability index (NDI). Notably, 60 of the participants (30 CON, 17 INP, and 13 TNP) performed the test on a second occasion to explore test-retest reliability. Results revealed a reduced *NormAcuity* for both INP and TNP compared with CON (*p* < 0.05). The neck pain groups had similar *Acuity* but longer *Time* compared with CON. Among TNP, there was a fair positive correlation between *Acuity* and NDI, while there was a negative correlation between *Acuity* and NDI among INP. Reliability measures showed good to excellent ICC values between tests, but standard error of measurements (SEM) and minimal detectable change (MDC) scores were high. The results showed that *NormAcuity* is a valuable measure to identify disturbed cervical movement sense among INP and TNP. While *Acuity* was similar between the groups, different strategies, such as longer *Time*, to perform the task among neck patient groups were used. Few differences were identified between the neck pain groups, but altered strategies may exist. Reliability was acceptable, and the test is feasible to perform in the clinic. However, the technical complexity of the automated image analysis is a concern. Future developments will provide more feasible solutions.

## Introduction

Neck pain accounts for a large part of total healthcare costs (1) and ranks high in terms of disability (2). Both factors stress the need for effective rehabilitation. Various symptoms besides pain can be associated with either trauma-induced or idiopathic neck pain. These include unsteadiness and visual disturbances, which can be attributed to altered cervical sensorimotor control (3). Multiple factors will contribute to persistent or recurrent symptoms with a recent opinion that unresolved disturbances in sensorimotor control may be a factor preventing full recovery (4, 5). Hence, measures of proprioceptive impairments are receiving more attention in neck pain and rehabilitation research (6–9).

Proprioception includes both conscious and unconscious awareness of joint position, movement, and force sense. Various methods using motion sensors have been used in research to reliably detect and quantify cervical joint position sense (JPS) (7) and movement sense deficits in individuals with neck pain (10–12). However, simple, cost-effective measures are needed in the clinical environment. Reliable and repeatable measures of JPS have been developed using a laser pointer projected onto a target (13), and this method has recently been adapted to measure cervical movement sense.

In this measure of movement sense, the subject traces a zigzag or a figure of eight patterns with a laser attached to the head. The performance is video-recorded, and the examiner determines the number of errors and time taken to perform the task. These measures are able to determine differences between individuals with neck pain and healthy participants (14). It was also found that the test demonstrates a clinically meaningful change in response to training (15) and the examiner's ratings of error and time taken were reliable (intra- and inter-tester) (16). These findings support the feasibility of these tracing tests for use in clinical practice (16). However, in some cases, the better outcome measure was the number of errors (14), while in others, it was the time taken when tracing the pattern (15). Thus, different strategies are employed to perform the task: the slower the time, few errors are noted, and the faster the time, the more errors are observed. A pilot analysis found that participants with neck trauma might have a different strategy than those with idiopathic neck pain (14), but this needs further investigation.

Röijezon et al. (17) recently tested a similar tracing method to objectively assess the proprioceptive capacity of the hand. Instead of human ratings of the performance, video recordings were analyzed with custom-made computer software to calculate movement acuity and speed (18). Acuity and speed add relevant information to the task performance (19) but are not possible to score with a human visual rating of the test. Therefore, this automatic video analysis might help to better understand strategies of performance of cervical movement sense between individuals with and without a traumatic onset of neck pain. Furthermore, an automated analysis would allow test-retest evaluation of an individual's performance of the task, removing possible human analysis errors. Feasible, reliable, and valid objective assessments of movement impairments are important in the clinical setting to guide interventions and to evaluate treatment effects.

Thus, the aim of the study was to investigate and compare outcomes of the clinical cervical movement sense test between individuals with traumatic and non-traumatic onset chronic neck pain and healthy controls using an automatic video-based scoring system. We hypothesized that movement sense would be poorer in the groups with neck pain as compared to healthy controls and that different strategies would emerge between the groups. Associations between movement sense and level of neck disability were also investigated. Test-retest reliability was explored to determine the repeatability of the test over two test performances.

## Materials and Methods

### Study Design and Setting

An observational cross-sectional and test-retest design was used to evaluate the nature of any disturbances, discriminative validity, and repeatability of the performance on the clinical test of movement sense measured with an automatic scoring tool. The Regional Ethical Review Board in Umeå approved the study (ref no. 2017/518-31) and all participants signed informed consent prior to participation.

Data were collected at three different physiotherapy clinics in Sweden by three experienced physiotherapists with an expertise in musculoskeletal rehabilitation. The assessment procedure was standardized, practiced, and piloted by all assessors prior to data collection. Two assessors (CB and AD) collected data two times from the same participants 1 h apart (sample 1), i.e., for both cross-sectional group comparison and the test-retest evaluation. One assessor (A-LL) collected data one time for cross-sectional group comparison (sample 2). During the test procedure, a standardized test protocol was used in order to ensure that the same instructions were given, and the same procedure was followed on each test occasion regardless of the test leader.

### Participants

The participants, working-aged women and men, were recruited as convenience samples by advertising at the physiotherapy clinics and surrounding areas. Participants with neck pain due to trauma to the head or neck, e.g., from a car accident, or a blow to the region, are referred to as the trauma-induced neck pain (TNP) group, while those with neck pain without relation to trauma are referred to as the idiopathic neck pain (INP) group. Healthy participants were recruited as a control (CON) group. In the sample size calculation for the cross-sectional study, we used data of Time on Target for the task with medium difficulty from a previous study investigating movement sense in groups of INP, TNP (all whiplash), and CON (11). A significance level of *p* = 0.05, 80% power, and a 2:1 ratio between CON and each of the neck pain groups gave a sample size of 38 CON and 19 INP, while a lower sample size was needed for a comparison between CON and TNP. As we planned for three groups and *post-hoc* analyses with Bonferroni compensation, we aimed for at least 50 participants in the control group and 25 in each of the NP groups for sufficient power. For evaluation of test-retest reliability, 50 participants have been suggested (20).

Inclusion criteria for INP and TNP were chronic neck pain with a pain duration of at least 3 months and a minimum NDI score of 12 (calculated as a percentage). Inclusion criteria for CON were no history of neck pain that they had sought treatment for or that had prevented normal life. All participants had to understand written and spoken Swedish and should be between 20 and 65 years of age. Exclusion criteria were those with a history of spinal surgery, vestibular pathology, visual impairments that could influence the test performance, severe mental illness, and evidence of any neurological or rheumatic disease.

### Assessments

A questionnaire was used to gain participant demographic data and information about their current neck pain, including whether the onset of pain was related to head or neck trauma or had an insidious onset; the length of history (months); and the average pain intensity over the previous week using a numeric rating scale (NRS) with 0 representing no pain and 10 representing worst imaginable pain (21). Participants with neck pain completed the NDI as a measure of self-assessed disability (22) and were calculated as a percentage from 0 to 100, where a higher score indicates worse disability. Physical activity level was measured by indicator questions developed by the National Board of Health and Welfare (23). This includes two questions. The first question asks about physical exercise on a level that makes you short winded, rated 1–6 as the amount of time during a regular week with 1 = 0 min and 6 > 120 min. The second question asks about physical activity in daily life that is not exercise and lasts for at least 10 min, rated 1–7 as the amount of time during a regular week with 1 = 0 min and 7 > 300 min.

**Figure 1:**
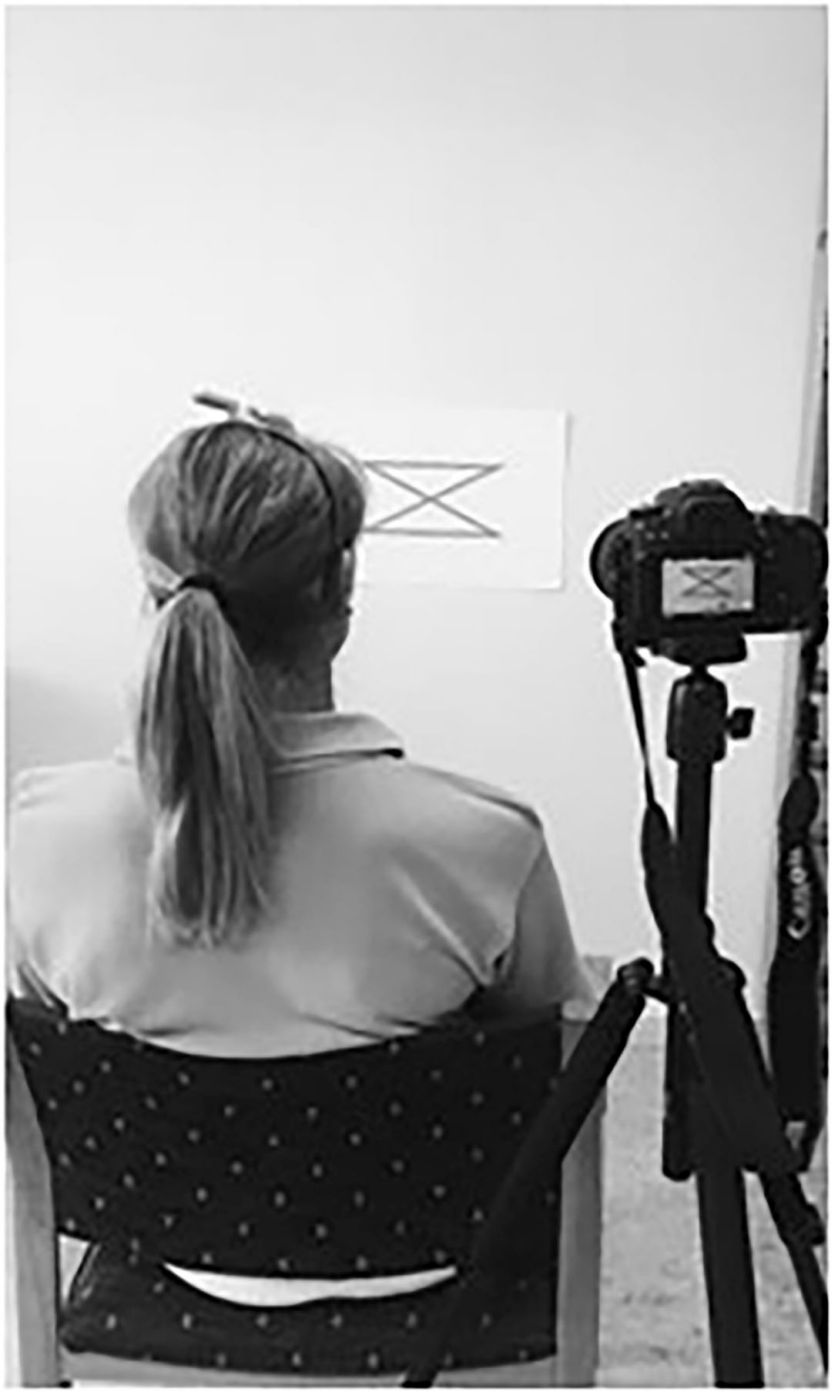
Cervical movement sense test. The test was performed in a corrected erect sitting posture with the head in neutral position and a laser pointer attached to the head. The task was to follow the target line as accurate as possible at a self-chosen speed with controlled head movements. The test was video-recorded for later automated image analysis for the extraction of outcome measures of the performance.

**Figure 2:**
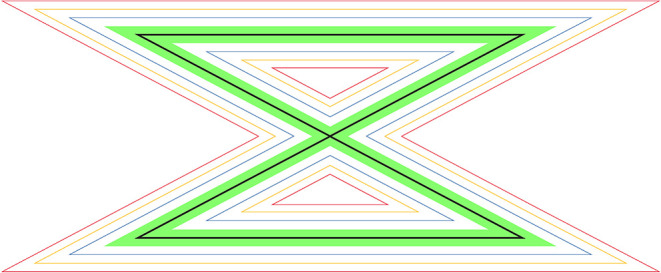
The target was a 1-mm thin black line at the center of 100 cm long zig-zag pattern printed on an A3 paper board.

A laser pointer fixed to the head *via* a headband was used in data collection for the movement sense test (Figure 1). The target was a 1 mm thin black line at the center of a 100 cm long zig-zag pattern printed on an A3 paper board (Figure 2). The participant sat in a relaxed, supported position, and the target pattern was attached to a wall, 100 cm from the laser pointer, such that the point of the laser dot was in the center of the pattern. To standardize this target position, the participant was instructed to close their eyes and sit quietly with their head in a neutral position, while the test leader positioned the center of the pattern on the laser dot on the wall. A digital video camera was affixed to a tripod and placed immediately behind and above the participant's right shoulder to record each trial for automated evaluation of the test performance.

The movement sense test was performed with the participant sitting erect on a stable chair with back support to minimize the influence of postural sway. The task was to trace the laser point along the black line, which was located in the middle of a horizontal and vertical zig-zag pattern, as accurately as possible by moving the head at a self-chosen speed (Figure 1). The task was performed in four different movement directions. It was first initiated from the left upper corner (left to right) and then from the right upper corner (right to left) with the zig-zag pattern in a horizontal plane. The pattern was then arranged in the vertical plane and first initiated from the top left corner (up to down) and finally from the bottom left corner (down to up). Three trials were performed for each direction, i.e., 12 trials were performed by each participant. For the repeatability, participants in sample 1 repeated this testing procedure 1 h after the initial test.

Each video recording was visually inspected to ensure that quality was satisfactory before automatic processing. A custom-made software for MATLAB was used to track the laser dot. It was developed to evaluate the movement sense of the hand (17). Detection of the four corners in the zigzag pattern, detection of the laser dot, and extraction of variables were conducted as previously described (18). Three different outcome variables were extracted from the video data as follows: *Acuity -* calculated as a percentage of the trial time the laser dot was on the black target line; *Speed*- average movement speed in mm/s; and *Time -* calculated in seconds to complete the task.

### Data Management and Statistical Analyses

Movement speed was self-chosen and not standardized in this study. This would lead to a well-known speed-accuracy trade-off (19). Hence, we calculated a fourth variable, normalized acuity (*NormAcuity)*, by dividing acuity with movement time. Data from the four different movement directions were pooled by calculating average scores leaving four outcome variables used in the statistical analyses, i.e., *Acuity, Speed, Time*, and *NormAcuity*.

Statistical analyses were performed using IBM SPSS Statistics, version 28, and Microsoft Excel 10. Histograms and Shapiro-Wilk tests were used to assess the normality of distribution for each variable. Outcome variables that were not normally distributed were logarithmically (Ln) transformed to meet the criteria for normal distribution. All analyses of the non-normally distributed data were conducted using the Ln transformed data, except for the calculation of standard error of measurement (SEM). The data presented in the tables are also untransformed for clearer interpretation.

Analyses of variance (ANOVA) was used for the assessment of group differences for each outcome variable. When the ANOVA showed a significant difference, *post-hoc* analysis was conducted with Bonferroni compensation between each of the three groups. The receiver operating characteristic (ROC) curve with area under the curve (AUC) was used to analyze sensitivity and specificity for a variable with a significant difference between the groups. Separate ROC and AUC were modeled for INP and CON and TNP and CON. Association between self-rated NDI and the movement sense variables was analyzed with Pearson correlation.

A two-way random-effects model with an absolute agreement and average measures was used to evaluate relative test-retest reliability ICC_2.k_. The ICC values were interpreted as follows: below 0.5 is poor reliability, between 0.5 and 0.75 is moderate reliability, between 0.75 and 0.90 is good reliability, above 0.9 is excellent reliability, and 1 is a perfect agreement (24). Paired *t*-tests were used to analyze whether systematic bias such as adaptation or learning effect occurred between test occasions 1 and 2. To evaluate absolute reliability, SEMs were calculated as the standard deviation of the difference between tests 1 and 2 divided by the square root of 2, and are presented as an indicator of the random error (noise in the data) between trials (20). Minimal detectable changes (MDCs), calculated as SEM × 1.96 × square root of 2, are also presented. The level of significance was established at *p* < 0.05.

## Results

Table 1 presents participant demographics for the cross-sectional and reliability studies.

**Table 1:** Characteristics of participants included in the cross-sectional and test-rest reliability analyses.

**Variable**	**CON**	**INP**	**TNP**	**Sign**
**Cross-section group comparison - sample 1 & 2**
N (female/male)	52 (41/11)	26 (21/5)	28 (22/6)	
Age (years)	46.9 (11.5)	49.5 (9.8)	45.6 (11.1)	0.425
Height (cm)	172.4 (7.0)	168.8 (10.7)	170.3 (9.6)	0.197
Weight (kg)	72.0 (14.9)	73.9 (14.7)	79.0 (17.3)	0.167
Physical activity (1–7)	6.0 (4.0; 6.0)	5.0 (4.0; 6.0)	5.0 (3.0; 6.0)	0.414
Physical exercise (1–6)	4.0 (2.3; 5.0)	3.0 (2.0; 5.0)	3.0 (1.3; 4.0)	0.158
NDI (0–100)	NA	29.9 (14.0)	40.5 (14.7)	0.009^a^
NRS pain (0–10)	NA	4.4 (2.6)	4.6 (2.5)	0.711^a^
Duration (months)	NA	100 (20)	154 (19)	0.057^a^
**Reliability evaluation - sample 1**
N (female/male)	30 (22/8)	17 (12/5)	13 (9/4)	
Age (years)	47.9 (9.5)	51.6 (9.3)	44.1 (10.6)	0.118
Height (cm)	173.8 (7.3)	171.0 (11.7)	173.2 (10.5)	0.622
Weight (kg)	74.0 (14.9)	73.5 (12.8)	83.6 (21.0)	0.151
Physical activity (1–7)	6.0 (4.0; 7.0)	6.0 (2.0; 5.0)	5.0 (3.0; 6.0)	0.243
Physical exercise (1–6)	4.5 (2.0; 6.0)	3.0 (2.0; 5.0)	3.0 (1.5; 4.5)	0.397
NDI (0–100)	NA	31.7 (14.4)	34.9 (10.4)	0.495^a^
NRS pain (0-−10)	NA	4.9 (2.2)	5.2 (1.8)	0.586^a^
Duration (months)	NA	83 (90)	144 (129)	0.096^a^

*Data are presented as frequencies, mean (±sd), or median (IQR1; IQR3)*.

a *Group comparison between INP and TNP*.

### Group Differences

Analyses (ANOVAs) revealed a significant difference between the groups for *NormAcuity* (Table 2). *Post-hoc* analyses indicated that *NormAcuity* was significantly reduced in INP (*p* = 0.023) and TNP (*p* = 0.023) compared with CON. There was a significant group difference for *Time* with *post-hoc* analyses revealing significantly longer movement time for TNP (*p* = 0.024) compared with CON. No significant differences were shown for *Acuity* or for *Speed* between the groups. There were no significant differences between INP and TNP in any measure.

**Table 2:** Group comparisons for the outcome variables of the videoed movement sense test.

**Variable**	**CON *n* = 52**	**INP *n* = 26**	**TNP *n* = 28**	**Sign**.	**Cohen's d**	**Power**
Acuity%	62.7 ± 1.7	64.2 ± 12.8	66.7 ± 13.7	0.400	0.018	0.206
Speed mm/s	61.7 ± 37.4	52.5 ± 32.3	46.0 ± 22.9	0.080	0.048	0.506
Time s	27.9 ± 11.5	37.2 ± 20.6	36.5 ± 13.3^*^	0.012	0.082	0.768
NormAcuity a.u.	2.7 ± 0.1	2.2 ± 1.1^*^	2.1 ± 0.6^*^	0.005	0.097	0.842

*Data are presented for the total group of samples 1 and 2 (n = 106). Ln-logged data were used for all variables except Acuity due to non-normal distribution for the ANOVAs, while non-logged data are presented in the table for clarity*.

* *p < 0.05 compared with the control group using Bonferroni*. *a.u., arbitrary unit*.

A ROC analysis on *NormAcuity* with NP (both INP and TNP in same group) and CON (*n* = 106) showed an AUC of 0.68, *p* = 0.02. A cutoff value of 2.5 gave a sensitivity of 0.72 and a specificity (i.e., false positive) of 0.46.

When doing separate ROC analysis for INP and CON, and TNP and CON, respectively, there are some slight differences with the advantage for TNP.

The ROC analysis on *NormAcuity* with INP (*n* = 26) and CON (*n* = 52) showed an AUC of 0.68, *p* = 0.02. The same cutoff value as described above, i.e., 2.5, gave a sensitivity of 0.65 and a 1-specificity of 0.48.

The ROC analysis on *NormAcuity* with TNP (*n* = 28) and CON (*n* = 52) showed an AUC of 0.69, *p* < 0.01. The same cutoff value as described above, i.e., 2.5, gave a sensitivity of 0.79 and a 1-specificity of 0.48.

### Association Between Self-Rated Disability and Movement Sense

There were no associations between NDI and the movement sense variables of *Speed, Time*, and *NormAcuity* in any group (Table 3). In the INP group, there was a fair negative correlation between NDI and *Acuity* (*r* = −373, *p* = 0.061) and a fair positive correlation between NDI and *Acuity* in the TNP group (*r* = 0.389, *p* = 0.041).

**Table 3:** Associations (Pearson correlations) between neck disability (NDI) and movement sense variables.

**Correlation NDI**	**Acuity %**	**Speed mm/s**	**Time s**	**NormAcuity a.u**.
INP *n* = 26	−0.373	0.179	0.017	−0.174
TNP *n* = 28	0.389^*^	−0.231	0.281	0.110

* *p < 0.05*. *a.u, arbitrary unit*.

### Reliability

Table 4 presents the ICC, SEM, and MDC for each variable. The ICCs ranged between 0.84 and 0.96. It is noted that there was a significant improvement for all outcome variables except *Acuity* at test occasion 2, indicating a systematic bias.

**Table 4:** Test-retest reliability for the movement sense variables.

**Variable**	**Test 1**	**Test 2**	**Sign between test 1 & 2**	**ICC**	**95% CI**	**SEM**	**MDC**
Acuity %	55.8 ± 8.5	55.3 ± 8.7	0.565	0.838	0.730–0.903	4.5	12.6
Speed mm/s	68.3 ± 37.5	78.4 ± 55.7	<0.001	0.960	0.914–0.979	17.3	48.0
Time s	27.8 ± 12.1	24.3 ± 12.8	<0.001	0.926	0.724–0.969	4.4	12.2
NormAcuity a.u.	2.4 ± 0.1	2.8 ±1.3	<0.001	0.913	0.727–0.962	0.4	1.1

*Intra-class correlation (ICC) is calculated as two-way random effects with absolute agreement and average measures. Data are presented for the total group of sample 1 (n = 60). Ln-logged data were used for all variables except Acuity due to non-normal distribution for the ICC analyses, while non-logged data are presented in the table for clarity and also used for calculation of standard error of measurement (SEM) and minimal detectable change (MDC)*.

*a.u, arbitrary unit*.

## Discussion

The results of the study suggest that automatized image analysis of the video recordings allows for a more in-depth analysis of the tracking performance, which is not available to the naked eye and may contribute to deeper information on performance strategies in persons with traumatic and non-traumatic neck pain compared with healthy individuals.

The cervical movement sense test in this study was performed and recorded similarly to that undertaken in previous studies (14–16). The difference was that we used an automatized image analysis pipeline for outcome measures of Acuity, Speed, and Time (17, 18) instead of a human visual assessment of the number of errors and time. *Time* should be similar between methods, as this relates to the time between the start and stop of the test. It is easily measured both visually and automatically from the videos. *Acuity* and *Speed* cannot be evaluated visually and are new measures in this study as is the combined measure of acuity and time *(NormAcuity)*, which accounts for any speed accuracy trade-off (17, 19, 25).

In relation to time, our participants took a longer time to complete the task. The participants with NP took 37 s and those with CON took 28 s compared with the 28 s and 23 s, respectively, as documented in a previous study by Ernst et al. (14). This difference may be due to variations in instructions or a difference in individual choice of movement speed between the study groups. Regardless of these possible variables and scoring methods, both studies found that participants with NP took a longer time to complete the task than CON participants.

Interestingly, the new variables *Acuity* (i.e., percentage of time on target) and *Speed* as single measures had limited value to discriminate between NP and CON. However, *NormAcuity* (*Acuity* divided by *Time*) provided a clearer picture of the performance between the groups. *NormAcuity* was the most useful variable to discriminate the neck pain groups (INP and TNP) from healthy controls. This demonstrates the need to consider accuracy and the time taken to perform the task collectively. This is logical, as the task was to be as accurate as possible with no restrictions regarding movement speed. According to Fitt's law, there is a negative association between movement speed and accuracy, the so-called speed-accuracy trade-off commonly reported (17, 19, 25). Hence, acuity tasks should either standardize the movement speed or as performed in this study *(NormAcuity)*, normalize acuity to movement time in the analyses for better discriminative ability between neck pain groups and healthy controls. In a similar way, the sum of the number of errors and time taken has been suggested as a possible solution to account for this when using human analysis of the test (15).

The ROC analyses of *NormAcuity* showed an AUC of approximately 0.7, which can be considered fair to the poor ability to discriminate between the groups. Using a cutoff value of 2.5 showed slightly better sensitivity in discriminating TNP vs. CON (0.79) compared with discriminating INP vs. CON (0.65). However, false positive (1-specificity) was high in both models (0.46).

We also determined that there are some different strategies between the groups. First, *Acuity* was slightly, but not significantly higher for INP and TNP compared with CON, while *Time* was longer for both the INP and TNP, which reached significance for TNP compared with CON. This finding indicates that the neck pain groups tended to prioritize accuracy over speed, while healthy controls were equally as accurate but faster in performing the task. Second, the correlation analyses showed potentially different strategies between the neck pain groups. A positive fair association between NDI and *Acuity* was seen in the TNP group, while the association (again fair) between NDI and *Acuity* was negative in the INP group. This indicates a higher *Acuity* within the TNP group with a higher (worse) disability, but lower *Acuity* within the INP group with a higher disability. The cause of this different movement behavior between the neck pain groups is difficult to speculate on and could be a random effect, although different strategies between those with INP and TNP have been seen in previous studies of balance and vertical perception (26, 27). A previous study reported a shorter *Time* among TNP compared with INP (14), but this was not confirmed in our study.

Evaluation of the test-retest repeatability (combining variance associated with the individual's performance and the automatic analysis) showed good to excellent ICC values for all outcome variables and was similar to that seen with previous inter- and intra-rater reliability of error and time analysis of the video of the same cervical movement sense test (16). Thus, participants performed the test in a similar manner on repeated occasions, although there was a systematic improvement on the second test occasion for all measures but *Acuity*. The SEM and the MDC must, however, be considered relatively large. According to these values, the NP groups in this study would have to exceed the performance of healthy CON to achieve an improvement according to the MDC for *NormAcuity*. Similar findings have been reported in previous research using a more unpredictable cervical movement sense test, although tested using Limits of Agreement instead of SEM and MDC (11). It could therefore be argued that it would be more clinically relevant to build a database with normative values of healthy people when evaluating clinically relevant improvements in clinical groups (11).

From a clinical standpoint, the results of this study add further support to the fact that sensorimotor functions are often disturbed in persons both with INP and TNP. The sensorimotor function assessed in this study was cervical movement sense and it was measured with technology that could become available in the clinic. Nevertheless, the video processing used in this study with the custom-made software was rather time-consuming and needs skills in MatLab or similar software. Therefore, at this time point, it is probably more feasible for most clinicians to use human visual rating of videos as previously described (14–16). Nevertheless, we contend that the automated measurement in this study, which allowed the measure of *acuity* and *speed*, is superior and feasible for the future with the rapid technological development with image analyses, sensors, and VR-technology. The future will see the development of tools with swift automated ratings for assessment and training [e.g., (11, 28)]. Such methods have the potential to provide objective assessments of movement impairments and various strategies between patients, which will be important guidance for tailored treatment interventions in line with precision medicine.

Some limitations need to be mentioned. First, in the traumatic group, we did not ask about the cause of trauma to the head or neck. Therefore, we do not know how many whiplash injuries were due to a car crash. Second, the test-retest reliability was measured with 1 h rest between test occasions. The reason for this rather short time period was to assure a steady condition between the tests, which is a challenge when using longer time periods, as neck pain is known to fluctuate over time (29). A risk of using only 1 h between test occasions is a fatiguing effect. However, this was not evident in this study, as all measures except *Acuity* improved on the second test occasion. This improvement indicates a systematic bias due to learning effects and needs to be considered if using the test as an evaluation of treatment effects pre- and post-intervention.

## Conclusion

Cervical movement sense measured as movement acuity normalized to movement time is disturbed in both INP and TNP groups. Neck pain groups prioritized movement acuity over speed, while healthy controls were faster but not more accurate. Test-retest reliability was good to excellent for the movement sense test using a laser pointer and videorecording, although high values were shown for SEM and MDC. Although the rating methods with automatized image analyses add an important value to the measure of the task performance, such as acuity and acuity, normalized to movement time, its clinical implication is still limited due to technical complexity compared with a human rating of the videos. Future innovations and research should evaluate new feasible and affordable technologies for reliable and valid assessment and tailored treatment methods.

## Data Availability Statement

The raw data supporting the conclusions of this article will be made available by the authors, without undue reservation.

## Ethics Statement

The studies involving human participants were reviewed and approved by Ethical approval was granted from the Swedish Ethical Review Committee (ref no 2017/518-31). The patients/participants provided their written informed consent to participate in this study.

## Author Contributions

CB and AD participated in the design of the study, were responsible for data collection of sample 1, performed initial analyses of the data, and contributed to writing the manuscript. UR was the project leader of the study and the main author of the manuscript, participated in the design of the study, and performed the final analyses of the data, including samples 1 and 2. PM participated in the design of the study, interpretation of the results, and contributed to writing the manuscript. GJ participated in the design of the study and had major contribution in discussing statistical analyses, interpretation, and writing the manuscript. JT participated in the design of the study and had major contribution in discussing statistical analyses, interpretation, and writing the manuscript. PK participated in the design of the study, created the software for automated data analyses of the videos, and extracted outcome measures used in the study. All authors read and approved the final manuscript.

## Conflict of Interest

The authors declare that the research was conducted in the absence of any commercial or financial relationships that could be construed as a potential conflict of interest.

## Publisher's Note

All claims expressed in this article are solely those of the authors and do not necessarily represent those of their affiliated organizations, or those of the publisher, the editors and the reviewers. Any product that may be evaluated in this article, or claim that may be made by its manufacturer, is not guaranteed or endorsed by the publisher.
